# Analysis of Differential Gene Expression under Acute Lead or Mercury Exposure in Larval Zebrafish Using RNA-Seq

**DOI:** 10.3390/ani14192877

**Published:** 2024-10-06

**Authors:** Xing Lu, Lang Zhang, Gen-Mei Lin, Jian-Guo Lu, Zong-Bin Cui

**Affiliations:** 1 Guangdong Provincial Key Laboratory of Microbial Culture Collection and Application, State Key Laboratory of Applied Microbiology Southern China, Institute of Microbiology, Guangdong Academy of Sciences, Guangzhou 510070, China; 2 Yangtze River Fisheries Research Institute, Chinese Academy of Fishery Sciences, Wuhan 430223, China; zhanglang@yfi.ac.cn; 3 School of Marine Sciences, Sun Yat-sen University, Zhuhai 519082, China; lingm5@mail.sysu.edu.cn (G.-M.L.); lujianguo@mail.sysu.edu.cn (J.-G.L.); 4 Southern Marine Science and Engineering Guangdong Laboratory (Zhuhai), Zhuhai 519080, China

**Keywords:** zebrafish, heavy metals, acute toxicity, RNA-seq, differential gene expression

## Abstract

**Simple Summary:**

Lead (Pb) and mercury (Hg) are two of the major heavy metals of antiquity and have gained considerable importance as potent pollutants in aquatic environments. It is known that fish embryos or larvae are more sensitive to the monitoring of heavy metal contamination. Thus, RNA sequencing (RNA-seq) analysis based on physiological changes in larval zebrafish was conducted to investigate the toxic mechanisms of lead or mercury in fish during early life stages. Our results showed that acute lead exposure significantly decreased survival but increased the malformation rates of developing zebrafish from 48 hpf to 120 hpf. Transcriptomic analysis revealed that lead-triggered biological processes included cellular process, metabolic process, biological regulation, and response to stimulus. The most enriched lead-regulated pathways included cytochrome P450, glutathione metabolism, and lipid metabolism. Moreover, a series of differentially expressed genes (DEGs) were identified by both mercury and lead treatment, which could be useful for searching potential molecular markers against the evaluation of heavy metals contamination.

**Abstract:**

This study was first conducted to investigate the effects of acute lead exposure on developing zebrafish embryos or larvae from 24 to 120 h post-fertilization (hpf). Our data showed that treatment with 50–200 μM lead significantly affected larval survivability and morphology compared to the respective control. Second, we chose 120 hpf larvae treated with 12.5 μM lead for RNA sequencing due to its exposure level being sufficient to produce toxic effects with minimum death and lead bioaccumulation in developing zebrafish. A total of 137.45 million raw reads were obtained, and more than 86% of clean data were mapped to the zebrafish reference genome. Differential expression profiles generated 116 up- and 34 down-regulated genes upon lead exposure. The most enriched GO terms for representative DEGs were ion transport and lipid metabolism. Third, a comparison with the dataset of mercury-regulated gene expression identified 94 genes (64 up-regulated and 30 down-regulated) for exposure specific to lead, as well as 422 genes (338 up-regulated and 84 down-regulated) for exposure specific to mercury. In addition, 56 genes were co-regulated by micromolar mercury and lead treatment, and the expression of thirteen genes, including *mt2*, *ctssb.1*, *prdx1*, *txn*, *sqrdl*, *tmprss13a*, *socs3a*, *trpv6*, *abcb6a*, *gsr*, *hbz*, *fads2,* and *zgc:92590* were validated by qRT-PCR. These genes were mainly associated with metal ion binding, proteolysis, antioxidant activity, signal transduction, calcium ion or oxygen transport, the fatty acid biosynthetic process, and protein metabolism. Taken together, these findings help better understand the genome-wide responses of developing zebrafish to lead or mercury and provide potential biomarkers for acute exposure to toxic metals.

## 1. Introduction

Heavy metals, such as lead (Pb) and mercury (Hg), are widely allocated in the human living environment and have great potential for biological toxicity and bioaccumulation [1]. These two toxicants exert a series of detrimental effects on organisms or cells through reactive oxygen species (ROS) production [2], physiological disruption [3], changes in certain enzymes or gene expression [4], and histological alterations [5]. Since lead and mercury are widespread in water environments on account of natural processes and anthropogenic activities [6], it is unavoidable for aquatic species to escape from the harmful influence of these pollutants. A large number of studies have characterized the toxicological effects of heavy metals on marine and freshwater fish, including hepatotoxicity [7], nephrotoxicity [8], neurotoxicity [9], reproductive toxicity [10], and endocrine disorders [11,12]. Recent research on adult zebrafish showed that short-time exposure (7 days) to 30 μg/L of lead acetate (PbAC) can cause liver metabolic disturbance and intestinal flora dysbiosis using the GC/MS metabolomics method [13]. Bakar et al. [14] found that embryonic exposure to 0.1 μM mercuric chloride (HgCl_2_) in zebrafish could increase locomotor activity in the dark, induce an imbalance of unsaturated fatty acids or amnio acid metabolites, and alter the expression of genes related to visual and behavioral impairments.

Zebrafish (*Danio rerio*) is an efficient animal model for developmental and toxicological research [15], which has many natural merits, such as small body size, in vitro fertilization, a short reproductive cycle, transparent embryos, as well as a low culturing cost. In addition, zebrafish and humans have more than 80% homology shared in their physiological changes and some molecular responses. Furthermore, zebrafish has abundant genetic resources that make it an ideal and cost-saving organism for RNA-seq investigation [16]. It is reported that embryo-larval (EL) toxicity tests are more accessible for the monitoring of heavy metal contamination [17]. Thus, to better understand the toxic mechanisms of lead and mercury on fish early development, RNA sequencing (RNA-seq) analysis was performed to investigate the transcriptional response of developing zebrafish to these two toxicants.

Although numerous studies have well documented the toxicity of lead or mercury on teleost fish, the molecular response mechanisms of fish embryo-larvae (EL) based on the transcriptomic level remain unclear. Moreover, our previous study reported that 391 up-regulated and 87 down-regulated genes were identified in developing zebrafish after acute exposure to micromolar mercury (0.1 μM). These genes can be further applicable for determining the robust molecular biomarkers used in the detection and assessment of mercury pollution. Therefore, in this study, we evaluated the effects of acute lead exposure on developing zebrafish embryos or larvae from 24 to 120 h post-fertilization (hpf). Some toxicological endpoints, including fish survivability, hatching success, and malformation, were examined to serve as forms of phenotypic anchoring for the subsequent transcriptomic analysis. The lead bioconcentration was also measured in larval zebrafish. High-throughput sequencing was then carried out to elucidate lead-induced transcriptional characterization in developing zebrafish. In addition, a comparison of gene expression profiles between lead- and mercury-treated transcriptomic data was performed in an attempt to search for crucial genes that function in the detoxification and excretion of mercury and lead or find common or novel molecular markers that respond highly to lead and mercury exposure.

## 2. Materials and Methods

### 2.1. Animals

Zebrafish (*Danio rerio*) derived from the AB strain were reared in an indoor recirculation system at a water temperature of 28.5 °C and pH of 7.0 ± 0.5, along with a photoperiod of 14 h light/10 h dark. Fish were hand-fed with live brine shrimp larvae twice daily (08:00 and 17:00). To achieve fertilized zebrafish embryos, adult males were held overnight in one aquarium with females separate. At the onset of light on the following morning, two males and one female were housed together in a 2 L plastic trap with a mesh bottom to prevent embryos from being eaten. Fresh eggs were obtained thirty minutes later and then placed in a thermostatic incubator with an embryo medium (19.3 mM NaCl, 0.23 mM KCl, 0.13 mM MgSO_4_·7H_2_O, 0.2 mM Ca(NO_3_)_2_, and 1.67 mM Hepes, pH 7.2). The embryonic development was staged in line with an hour post-fertilization (hpf) or standard histological features [18].

### 2.2. Toxic Lead Exposure on Fish

Acute toxicity assays were conducted to determine the physiological changes in developing zebrafish under different concentrations of lead exposure. A batch of 300 fresh embryos that developed normally for up to 24 hpf were chosen and then separated into 60 embryos/dish. The lead nitrate stocks (20 mM of Pb(NO_3_)_2_) were made and diluted using embryo medium to nominal concentrations for the exposure assay. According to our preliminary results of acute toxicity on zebrafish embryos, the minimum and maximum concentrations of Pb(NO_3_)_2_ ranged from 12.5 μM to 200 μM for 96 h of exposure. Thus, fish embryo-larval were exposed from 24 hpf to 120 hpf at 28 °C in water medium containing 0 (control), 12.5, 25, 50, 100, and 200 μM of Pb(NO_3_)_2_, respectively. Three replicates were performed from each treatment.

During the exposure period, the culture solution was renewed every 12 h. Dead or moribund fish were under consideration for unstable swimming actions, no reaction to stimulus, and no heartbeat. Fish survival, hatching success, and malformation (e.g., pericardial edema and axial spinal curvature) at the corresponding developmental stages were recorded under a stereomicroscope (Carl Zeiss, Oberkochen, Germany). After 96 h of exposure, zebrafish larvae at the 120 hpf stage were randomly collected for further analysis from each exposure group (n = 3). A portion of larvae was used for lead contents, and others were soaked in RNAstore reagent (Beyotime, Shanghai, China) for gene expression analysis.

### 2.3. The Measurement of Lead Concentrations

Lead levels accumulated in zebrafish larvae were determined using the atomic absorption spectrometry (AAS, Varian AA240, Varian, Victoria, Australia) and converted as μg/g dry mass, as reported previously [19].

### 2.4. RNA Extraction

Depending on the results of survivability and bioaccumulation from acute lead exposure, larval zebrafish at the 120 hpf stage were collected from the control group and 12.5 μM from the lead treatment group, respectively. Total RNAs were extracted from developing zebrafish using the TRIZOL reagent (Invitrogen, Carlsbad, CA, USA), following the manufacturer’s instructions. RNA quantity and integrity were assessed using NanoDrop 8000 (Thermo Scientific, Waltham, MA, USA) and 1.5% of agarose gel electrophoresis.

### 2.5. Illumina Sequencing

RNA library preparation and high-throughput sequencing were performed by experts at the Beijing Genome Institute of China (BGI, https://www.bgi.com/, accessed on 4 September 2024). The sequencing library was constructed and then pair-ends-sequenced for 2 × 100 bp on an Illumina HiSeq^TM^ 2000, as described previously [20].

### 2.6. Bioinformatic Analysis

Briefly, the obtained raw data were first filtered by the FASTX-Toolkit to eliminate poor reads (including adaptors, quality score < 5, and ambiguous bases). Then, the clean reads were generated and mapped to the zebrafish reference genome (*Danio_rerio* Zv9.72 downloaded from Ensembl database) using TopHat (v.2.0.9). The read calculations and sorting of the alignment files were conducted by SAMtool (v.0.2.0), and then assembled into transcripts through Cufflinks (v. 2.0.2). The abundance of assembled transcripts was calculated as FPKM (fragments per kilobase per million) value. The read counts were merged into each feature [21] and subsequently used for differentially expression analysis using Cuffdiff with default settings. Genes that met the cut-off criterion of fold change ≥ 2 and *p*-values < 0.05 were regarded as significantly or differentially expressed. GO and KEGG enrichment analyses of differentially expressed genes (DEGs) were identified using WeGO [22] and ClueGO programs [23], respectively. The functional databases for GO and KEGG were accessed as described in our previous publication [24].

### 2.7. Validation Analysis via Quantitative Real-Time PCR (qRT-PCR)

The qRT-PCR assay was performed according to the MIQE-compliant guidelines. First-strand cDNA was synthesized from DNase I-treated RNA using the RevertAid^TM^ First Strand cDNA Synthesis Kit (Fermentas, Hanover, NH, USA). The Primer Premier 6.0 was used to design the primers of candidate genes and were further analyzed by a Primer-BLAST tool. qRT-PCR was conducted in a CFX Connect^TM^ Real-Time PCR Detection System (BioRad, Hercules, CA, USA). The amplification was conducted at a 20 μL volume consisting of a 10 μL 2× iQ SYBR Green Supermix, 0.4 μL PCR forward/reverse primers (10 μΜ), 5 μL 10× diluted cDNA templates, and 4.2 μL nuclease-free water. Three independent parallels were performed in the analysis, and each qRT-PCR reaction was conducted in biological triplicates. Thermos cycling parameters were set at 95 °C for 1 min, followed 40 cycles comprising 95 °C for 10 s, 60 °C for 30 s, and 72 °C for 10 s. Melting curve analysis was conducted by heating from 65 °C to 95 °C with an increase of 0.5 °C. The specificities of primer sequences were confirmed by a sole melt peak and the predicted amplicons (bp) on agarose gel.

Prior to the qRT-PCR experiment, the primer curves were obtained by a regression of the Cq value using serial 5-fold cDNA dilutions from the mixture of 120 hpf larvae. Amplification efficiency was calculated by the formula of E% = (10^−1/slope^ − 1) × 100. qRT-PCR information, including GenBank accession number, primer sequence, and amplification efficiency, as well as the amplicon length, are all displayed in Table S1. The housekeeping gene *β-actin* was utilized as an internal reference due to there being no alternations in RNA-seq analysis for lead treatment. Moreover, *β-actin* was also reported in zebrafish for the normalization of heavy metal-induced gene expressions based on previous reports [25]. For quantification, the 2^−ΔΔCt^ method was used to analyze the relative copy numbers of candidate genes [26].

### 2.8. Comparison of Lead and Mercury Exposure Transcriptomic Data in Zebrafish Larvae

In the present study, we compared micromolar mercury treatment with lead treatment transcriptomic data in larval zebrafish at 120 hpf in order to characterize the transcriptional events co-regulated by these two toxic metals. As described in our previous study [20], zebrafish embryos were treated with 0.1 μM mercuric chloride (HgCl_2_) from 24 hpf to 120 hpf. The exposure conditions and time were the same as in acute lead toxicity. These sequencing data were downloaded from the NCBI Sequence Read Archive under accession number SRP089827. Notably, the exposure concentration for mercury was determined based on the survival rate, which increased slightly but insignificantly at 0.1 μM, which was, however, able to induce the obvious bioaccumulation.

### 2.9. Statistical Analysis

Data are shown as mean ± standard deviation (SD), and SPSS 26.0 software was used for statistical analysis. Differences in the data of fish survival, hatching success, malformation, and metal bioaccumulation between control and experimental groups were analyzed by one-way analysis of variance, followed by Duncan’s post hoc test. Differences in the data of gene expression among different treatments were assessed by an independent samples *t*-test. The correlation between the results from RNA-seq and qRT-PCR was estimated by Spearman’s rho test. Statistical significance was set at *p* < 0.05.

## 3. Results

### 3.1. Effects of Lead Exposure on Zebrafish Development and Lead Bioaccumulation in Larval Zebrafish

**Figure 1:**
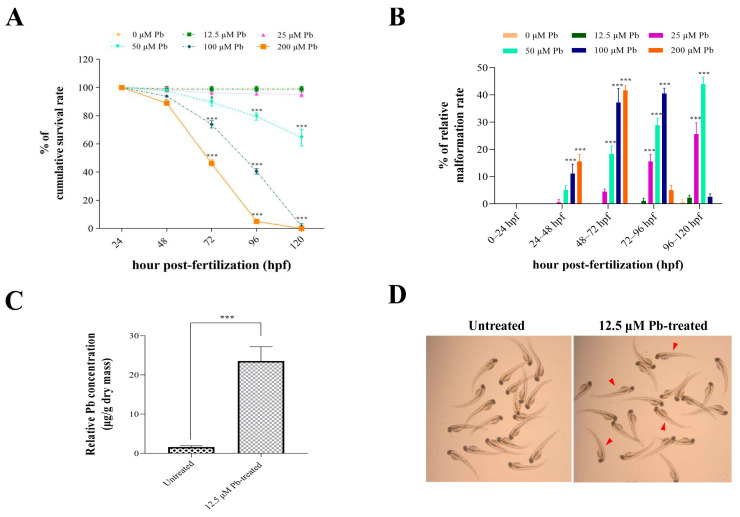
Physiological changes in developing zebrafish in response to acute lead exposure from 24 hpf to 120 hpf. (**A**) Survival rate of developing zebrafish after 96 h of exposure to nominal lead concentrations of 12.5 μM, 50 μM, 100 μM, and 200 μM, respectively. (**B**) Malformation rate of developing zebrafish after 96 h of exposure to lead. (**C**) Bioaccumulation in zebrafish larvae at 120 hpf after treatment with 12.5 μM lead; (**D**) Morphology of larval zebrafish at 120 hpf after treatment with 0 (control) and 12.5 μM lead for 96 h. Red arrowheads indicate representative normal larvae. Values represent the mean ± standard deviation of three biological replicates. * *p* < 0.05; *** *p* < 0.001.

First, we explored the effects of acute lead exposure on physiological responses of developing zebrafish embryos from 24 hpf to 120 hpf. As shown in Figure 1A, the survival rates of developing embryos from the 72 hpf to 120 hpf stage significantly decreased after exposure to lead at concentrations of 50–200 μM, while no significant differences were determined for larvae hatching success. Second, the malformation rates of larvae from 48 hpf to 120 hpf marginally increased from 11.11% to 43.89%, with an increase in lead concentrations from 25 to 200 μM (Figure 1B). Third, the lead bioaccumulation in the 12.5 μM lead-exposed group was significantly higher than that in the untreated control (Figure 1C). Furthermore, treatment with 12.5 μM lead produced over 95% larvae survivability with no obvious malformations (Figure 1D). Based on these data, we chose 12.5 μM lead following RNA-seq due to this exposure level being sufficient to produce toxic effects with minimal death and lead bioaccumulation in zebrafish larvae.

### 3.2. Lead-Regulated Gene Expression

RNA sequencing produced 51.43–67.33 million (M) pairs of clean reads in this study (Table 1). About 58.41 M (86.75%) with 49.37 M (73.32%) unique reads for the control, and 44.69 M (86.88%) with 37.94 M (73.76%) reads for lead treatment were aligned to the zebrafish reference genome. A similar RPKM distribution was found in both groups (Figure S2), and a total of 18,499 genes with a mean abundance > 0.1 FPMK were finally considered to be expressed (Table S2).

**Figure 2:**
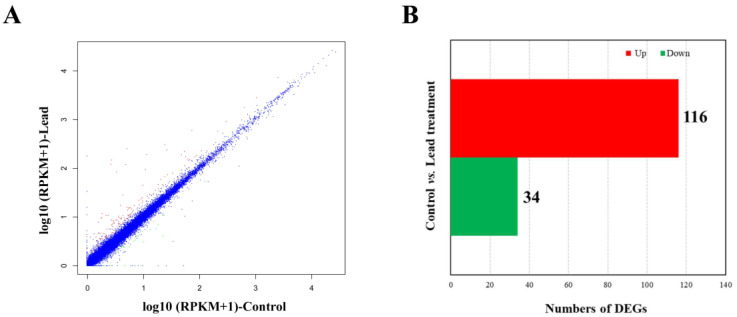
Bioinformatic analysis of RNA-seq data. (**A**) Correlation of gene expression between untreated control and lead-treated groups. Red and green dots indicated up- and down-regulated genes, respectively. The blue dot referred to no significantly expressed genes. (**B**) Numbers of differentially expressed genes between control and lead treatments. Expression differences are displayed in different colors. Red indicates up-regulated genes, and green shows down-regulated genes.

Genes with a fold change ≥ 2 and *p*-value < 0.05 were defined as differentially expressed genes (DEGs) (Figure 2A). Table S3 and Figure 2B display the 116 up-regulated and 34 down-regulated genes, respectively. Genes such as *lepa* (leptin a), *lepb* (leptin b), and *mib2* (mindbomb E3 ubiquitin protein ligase 2) were specifically expressed in lead-exposed embryos. Genes including *slc2a9l1* (solute carrier family 2, member 9-like 1), *mt2* (metallothionein 2), *ctssb.1* (cathepsin Sb, tandem duplicate 1), *slc2a11l* (solute carrier family 2 (facilitated glucose transporter), member 11-like), and *tcap* (telethonin) were among the top lead-induced DEGs, while *trps1* (trichorhinophalangeal syndrome I), *fads2* (fatty acid desaturase 2), *gck* (glucokinase (hexokinase 4)), *helt* (helt Bhlh transcription factor), and *sqlea* (squalene epoxidase a) were the most highly inhibited following lead exposure. Other uncharacterized DEGs are also displayed in Table S3.

### 3.3. The Confirmation of RNA-Seq Data with qRT-PCR

**Figure 3:**
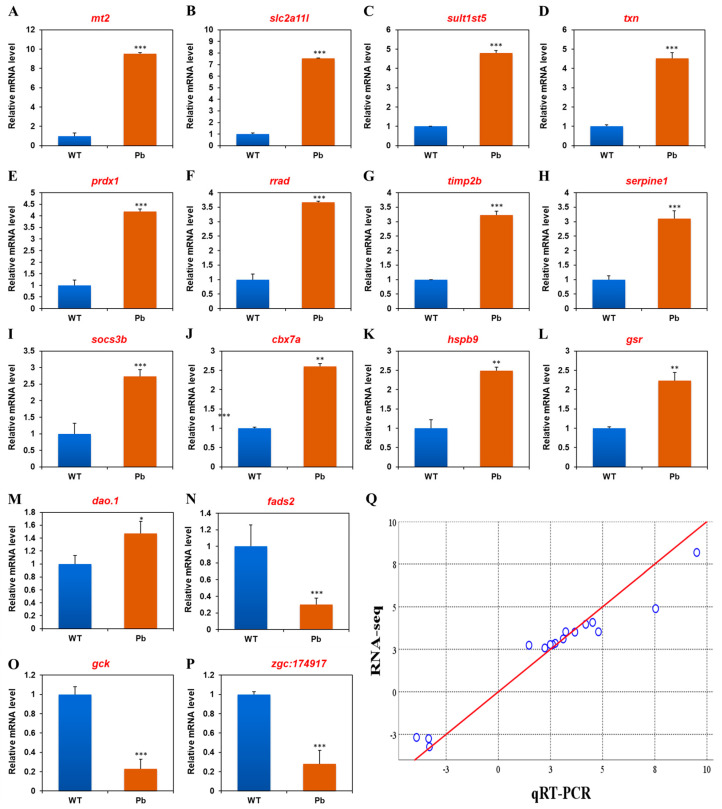
qRT-PCR experiment validated the expression of sixteen DEGs, including *mt2* (**A**), *slc2a11l* (**B**), *sult1st5* (**C**), *txn* (**D**), *prdx1* (**E**), *rrad* (**F**), *timp2b* (**G**), *serpine1* (**H**), *socs3b* (**I**), *cbx7a* (**J**), *hspb9* (**K**), *gsr* (**L**), *dao.1* (**M**), *fads2* (**N**), *gck* (**O**), and *zgc:174917* (**P**). (**Q**) Scatterplots for fold change in gene expressions were performed by RNA-seq versus qRT-PCR results. The straight reference line in red indicates a linear relationship between the data of RNA-seq and qRT-PCR (*p* < 0.01, correlation coefficient = 0.971). Error bars represent the standard deviation of three biological replicates. * *p* < 0.05; ** *p* < 0.01; *** *p* < 0.001.

To validate the RNA-seq results, sixteen genes from the DEGs dataset were measured with qRT-PCR. The standard curves for these primer pairs are first evaluated in Figure S1. Next, the expression pattern shows good similarity for both up- and down-regulated genes (Table 2). Furthermore, Spearman’s rho analysis reveals that the data of RNA-seq and qRT-PCR are significantly correlated (*p* < 0.01, correlation coefficient = 0.971, Figure 3), indicating the reliability of RNA-seq data.

### 3.4. Functional Classification of Lead-Regulated DEGs

**Figure 4:**
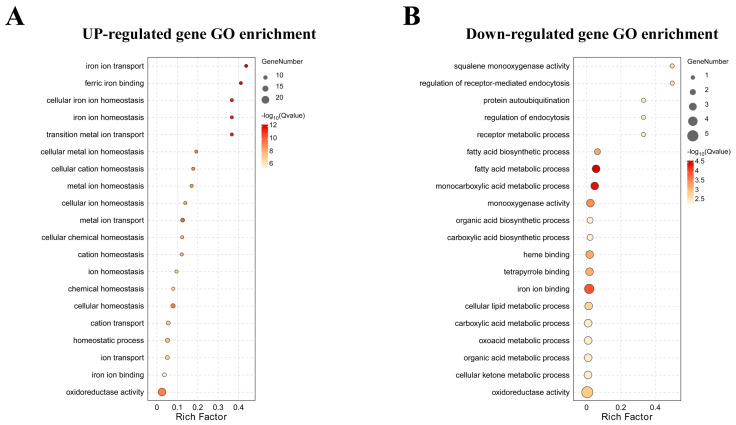
GO term enrichment of significant DEGs in untreated control and lead-treated groups. Bubble plots indicate enriched GO terms in the up-regulated (**A**) and down-regulated (**B**) genes. The Y axis denotes GO terms, whilst the X axis designates the rich factor. Rich factor refers to the ratio of DEGs relative to all genes subjected to GO annotation, and the higher the rich factor, the greater the intensity. The input number indicates the number of DEGs enriched in a specific GO term.

GO enrichment analyses indicated that 135 DEGs with enriched GO terms were grouped into 6 ‘cellular component’, 9 ‘molecular function’, and 14 ‘biological processes’ (Figure S3). Among these ‘biological processes’ categories, genes that were up- and down-regulated by lead exposure, mainly populated the cellular process, metabolic process, biological regulation, and response to stimulus. All DEGs associated with enriched GO terms are displayed in Table S4. As shown in Figure 4, the enriched GO terms for lead-inducible genes were ion transport, binding, and homeostasis. In contrast, several specific processes, such as fatty acid metabolic and biosynthetic process, were significantly enriched by lead-inhibited genes.

Table 3 shows the top 5 pathways resulting from KEGG enrichment, and a total of 101 pathways were determined by lead treatment (Table S5). Up-regulated DEGs were mainly enriched in the metabolism of xenobiotics by cytochrome P450, glutathione metabolism, drug metabolism–cytochrome P450, complement and coagulation cascades, and adipocytokine signaling pathway, whereas the down-regulated DEGs were grouped into steroid biosynthesis, sesquiterpenoid and triterpenoid biosynthesis, as well as butirosin and neomycin biosynthesis. These pathways are primarily involved in the regulation of biological processes, such as the metabolic process, biosynthesis process, and signal transduction.

### 3.5. Genes Regulated by Mercury and Lead Exposure

**Figure 5:**
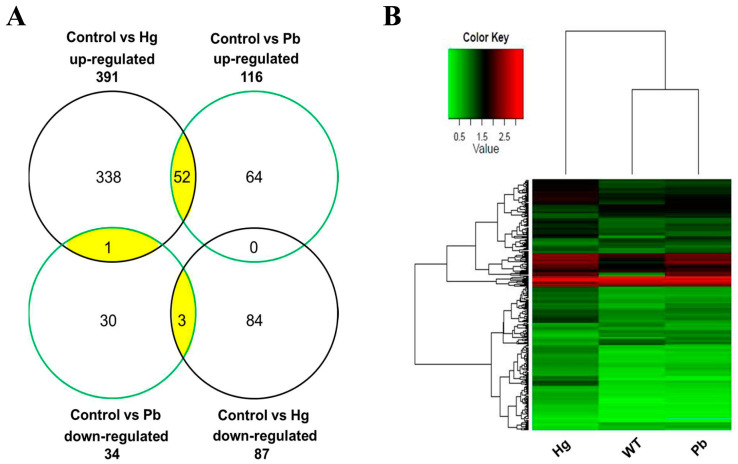
Gene expression profiling in larval zebrafish upon micromolar mercury and lead exposure. (**A**) Venn diagrams represent the number of up-regulated and down-regulated genes. Genes that change their expression in zebrafish larvae exposed to mercury are shown in a black circle. Genes that change their expression in zebrafish larvae exposed to lead are displayed in a green circle. Genes that are commonly regulated by mercury and lead are shown in a yellow oval. (**B**) Hierarchical clustering analysis based on gene expression profiles. Red shows up-regulated expression, while green represents down-regulated expression. Each column indicates different treatment groups, and each horizontal line refers to a gene. The color scale shows fold changes in gene expression.

A comparison with our previous mercury (0.1 μM HgCl_2_)-regulated gene expression dataset [20] was performed to characterize the co-transcriptional events under lead and mercury treatment. As shown in Figure 5A and Table S6, there were 52 up-regulated and 3 down-regulated genes in both the lead and mercury exposure group, respectively; however, one uncharacterized gene (*si:ch211-284o19.8*) was up-regulated in the mercury exposure group but down-regulated in the lead exposure group. Hierarchical clustering analysis showed that different heavy metal treatments resulted in distinct gene expression patterns (Figure 5B). In addition, 64 up-regulated and 30 down-regulated genes for acute exposure specific to lead, as well as 338 up-regulated and 84 down-regulated genes for acute exposure specific to mercury were identified. Several representative genes, including *intl2* (intelectin 2), *per2* (period circadian clock 2), *cry5* (cryptochrome circadian clock 5), *cybb* (cytochrome *b*-245, beta polypeptide), *hamp1* (hepcidin antimicrobial peptide), *nyx* (nyctalopin), and *opn1sw1* (opsin1 (cone pigments), short-wave-sensitive 1), for exposure specific to mercury, and *sult1st5* (sulfotransferase family 1, cytosolic sulfotransferase 5), *rrad* (Ras-related associated with diabetes), *socs3b* (suppressor of cytokine signaling 3b), *hspb9* (heat shock protein, alpha-crystallin-related, 9), and *gck* (glucokinase) for exposure specific to lead displayed different expression patterns in developing zebrafish (Table 4).

The expression of thirteen genes (eleven up-regulated and two down-regulated) were investigated using qRT-PCR assays to validate their co-regulation upon lead and mercury exposure. As shown in Table 4, the expressions of *mt2* (metallothionein 2), *ctssb.1* (cathepsin Sb, tandem duplicate 1), *prdx1* (peroxiredoxin 1), *txn* (thioredoxin), *sqrdl* (sulfide quinone reductase-like), *tmprss13a* (transmembrane protease, serine 13a), *socs3a* (suppressor of cytokine signaling 3a), *trpv6* (transient receptor potential cation channel, subfamily V, member 6), *abcb6a* (ATP-binding cassette, sub-family B (MDR/TAP), member 6a), *gsr* (glutathione reductase), and *hbz* (hemoglobin zeta) were induced by both mercury and lead stress, while the expressions of *fads2* (fatty acid desaturase 2) and an uncharacterized gene *zgc:92590* were inhibited by mercury and lead exposure. Moreover, searching against the gene ontology database revealed that co-up-regulated DEGs are associated with metal ion binding, proteolysis, antioxidant or oxidoreductase activity, signal transduction, and calcium ion or oxygen transport, while the function annotations for co-down-regulated genes include the fatty acid biosynthetic process and protein metabolism (Table 4).

## 4. Discussion

In the present study, we first investigated the physiological changes in developing zebrafish embryos after 96 h of exposure to toxic lead. The larvae at the 120 hpf stage showed higher mortality and malformation rates when treated with lead at concentrations of 50–200 μM. This is illustrated by the fact that lead may induce the deregulation of essential ion homeostasis (e.g., calcium) or defection in the myotomes of somites [27]. As the lead exposure level increased from 100 μM to 200 μM, over 95% of zebrafish larvae were found dead at the 96 hpf and 120 hpf stage, which might be associated with the disturbance in structure and function during embryonic development or the inhibition of enzyme synthesis and activity [17].

Subsequently, RNA-seq identified a total of 150 genes (116 up- and 34-down-regulated genes) in zebrafish larvae after acute exposure to 12.5 µM lead for 96 h. Significant lead-inducible genes include feed intake (*lepa* and *lepb*; see the NCBI official full name in Table S3), embryonic growth and development (*vtg7*), immune response (*socs3a*, *socs3b*), and lead-inhibited lipid metabolism (*fads2*, *sqlea*). Lepa and Lepb were identified as leptin orthologs in both diploid and tetraploid fish species [28,29]. Most studies on teleost fish indicate that leptin is mainly involved in the regulation of feed intake and energy balance [30,31]. The up-regulated expression of *lepa* and *lepb* may suggest that toxic lead exerts neurotoxic effects on appetite control in zebrafish early development. Vtg7 was characterized as a member of vitellogenins that function as precursors for egg-yolk proteins [32]. The up-regulation of this gene underlies enhanced larval resistance to micromolar lead treatment as a result of nutrients supply. It has been reported that *socs3* gene expression is regulated by immune stimulation in teleost [33], and it is involved in controlling the signaling of cytokines and hormones that affect immunity and growth [34]. The up-regulation of *socs3a* and *socs3b* implies that zebrafish are actively protected from potentially lead-induced immune system damage. In addition to those up-regulated genes, the down-regulation of *fads2* and *sqlea* was detected and associated with fatty acid biosynthetic and the metabolic process, which is ascribed to the developmental toxicity observed in lead-exposed zebrafish larvae. Moreover, genes such as metallothionein (*mt2*), peroxiredoxin (*prdx1*), heat shock proteins (*hspb9*, *hspb11*), thioredoxin/glutathione reductase (*txn*, *gsr*), and glutathione S-transferase (*gsto1*, *gstp1*) are largely reported to be associated with antioxidant response [35]. For instance, metallothionein is a small, cysteine-abundant, and metal-binding protein that can become involved in a series of stress responses [36]. Kim et al. [37] reported that dietary lead exposure at 60 mg/kg significantly stimulated metallothionein gene expression in Korean Rockfish (*Sebastes schlegelii*). Heat shock proteins (HSPs) are highly conserved and are normally major indicators to measure the stress response of fish exposed to toxicants [38]. Recent research indicated that waterborne lead exposure at 50–800 μg/L significantly enhanced expressions of HSP genes in snakehead (*Channa argus*) [39]. Thus, the up-regulation of these genes may indicate a reduction in lead-triggered oxidative stress in fish. These data might extrapolate toxicogenomic data and contribute to more in-depth research on the molecular mechanisms of lead toxicity in early fish life stages.

GO enrichment analysis revealed that the most enriched biological processes for genes either up-regulated or down-regulated by lead were ion transport and lipid metabolism, respectively (Table S4). The ion transport induced by lead may be related to the function of lead metal ions to replace some bivalent cations, which ultimately disturb the biological metabolism of the cell [40]. Lipids can supply fundamental energy for developing fish [41], and the down-regulation of lipid metabolism-related genes suggests that there is a reduced capacity for energy supplementation upon lead stress. These data are consistent with the report by Xia et al. [13], who concluded that short-term lead exposure for adult zebrafish could significantly affect liver glucose and lipid metabolism. After KEGG enrichment analysis, the most abundant up-regulated pathways included cytochrome P450 and glutathione metabolism. These two pathways are linked to the xenobiotic metabolism and detoxification process. Cytochrome P450 is responsible for the metabolic activation of the xenobiotics and facilitates the body to efficiently eliminate various toxicants [42]. The induction of the cytochrome P450 system consolidates the capability of developing embryos/larvae in lead detoxification. Furthermore, the presence of this key function of detoxification is further supported by the high induction of glutathione metabolism, which is known to function in detoxifying toxic metals [43]. On the contrary, several biosynthesis pathways, such as steroid or unsaturated fatty acids biosynthesis, were found to be enriched in the down-regulated pathways. These results are in agreement with the notion that acute lead exposure has adverse effects on lipid metabolism in larval zebrafish. Hence, enriched biological processes or pathways are important physiological alterations for developing zebrafish upon acute lead tress.

A comparison with the dataset of mercury-regulated gene expression identified 56 genes co-induced by micromolar mercury (0.1 μM) and lead treatment (12.5 μM), and the reliability of RNA-seq results was confirmed by the qRT-PCR analysis of thirteen selected genes. Among these DEGs, *ctssb.1* (cathepsin Sb, tandem duplicate 1) and *tmprss13a* (transmembrane protease, serine 13a) were related to proteolysis, which can remove abnormal or damaged proteins from the cell under metal-induced oxidized stress [44]. Sqrdl (sulfide quinone reductase-like) has important roles in catalyzing various sulfides to persulfide [45], thus lowering cellular sulfide levels under xenobiotic stress. Trpv6 (transient receptor potential cation channel, subfamily V, member 6) has been shown to be a main Ca^2+^ transporter identified in human [46], which mediates the transport of several heavy metals such as zinc and cadmium. Gsr (glutathione reductase) is responsible for converting oxidized glutathione (GSSG) into reduced glutathione (GSH), which has a protective effect against oxidative injury. Hbz (hemoglobin zeta) belongs to the hemoglobin family and can exhibit strong sensitivity to heavy metal exposure [47]. These DEGs co-treated by mercury or lead are also found in other aquatic species or plants and thus might serve as early biomarkers for an environmental risk assessment.

## 5. Conclusions

In conclusion, we have investigated the effects of lead exposure on physiological changes and bioaccumulation in zebrafish embryo-larvae. RNA-seq identified 116 up- and 34 down-regulated genes in 120 hpf larval zebrafish after exposure to 12.5 μM lead for 96 h. Lead-triggered biological processes included the cellular process, metabolic process, biological regulation, and response to stimulus. The most enriched lead-regulated pathways included cytochrome P450, glutathione metabolism, and lipid metabolism. Moreover, a comparison with the dataset of mercury-regulated gene expression identified 94 genes (64 up-regulated and 30 down-regulated) for exposure specific to lead, as well as 422 genes (338 up-regulated and 84 down-regulated) for exposure specific to mercury. In addition, 56 genes were co-regulated by micromolar mercury (0.1 μM) and lead treatment (12.5 μM). Many DEGs could be useful for researching potential molecular markers against the evaluation of heavy metal contamination. Collectively, our data provide insights into the genome-wide transcriptional alteration of developing zebrafish underlying acute heavy metals exposure.

## Figures and Tables

**Table 1:** Statistics for read mapping.

Group Name	Control	Control%	Lead	Lead%
Total reads	77,597,408	100.00%	59,858,446	100.00%
Total bases pairs	7,682,143,392	100.00%	5,925,986,154	100.00%
Processed reads	67,329,394	86.77%	51,432,756	85.92%
Processed bases pairs	6,665,610,006	86.77%	5,091,842,844	85.92%
Low-quality reads	6,769,040	8.72%	5,304,506	8.86%
Adapter polluted reads	3,498,974	4.51%	3,121,184	5.21%
Total mapped reads	58,407,461	86.75%	44,685,271	86.88%
Unique mapping	49,365,912	73.32%	37,937,786	73.76%
Total unmapped reads	8,921,933	13.25%	6,747,485	13.12%

**Table 2:** Comparisons between RNA-seq data and qRT-PCR results.

Gene Symbol	Gene Name	Fold Change (Pb)	
		RNA-Seq	qRT-PCR
*mt2*	metallothionein 2	8.18	9.51 ± 0.13
*slc2a11l*	solute carrier family 2 (facilitated glucose transporter), member 11-like	4.89	7.54 ± 0.03
*sult1st5*	sulfotransferase family 1, cytosolic sulfotransferase 5	3.53	4.80 ± 0.12
*txn*	thioredoxin	4.08	4.52 ± 0.30
*prdx1*	peroxiredoxin 1	3.97	4.19 ± 0.11
*rrad*	Ras-related associated with diabetes	3.51	3.67 ± 0.03
*timp2b*	TIMP metallopeptidase inhibitor 2b	3.53	3.23 ± 0.13
*serpine1*	serpin peptidase inhibitor, clade E (nexin, plasminogen activator inhibitor type 1), member 1	3.11	3.11 ± 0.26
*socs3b*	suppressor of cytokine signaling 3b	2.85	2.73 ± 0.21
*cbx7a*	chromobox homolog 7a	2.77	2.60 ± 0.08
*hspb9*	heat shock protein, alpha-crystallin-related, 9	2.78	2.49 ± 0.09
*gsr*	glutathione reductase	2.58	2.23 ± 0.22
*dao.1*	D-amino-acid oxidase, tandem duplicate 1	2.74	1.47 ± 0.19
*fads2*	fatty acid desaturase 2	−2.75	−3.34 ± 0.08
*gck*	glucokinase	−2.68	−3.92 ± 0.10
*zgc:174917*	uncharacterized gene	−3.23	−3.30 ± 0.14

**Table 3:** Top 5 pathways from KEGG enrichment for up- and down-regulated genes.

KEGG Pathways for Up-Regulated Genes		KEGG Pathways for Down-Regulated Genes	
Pathway Name	*p*-Value	Pathway Name	*p*-Value
Metabolism of xenobiotics by cytochrome P450	1.02 × 10^−5^	Steroid biosynthesis	0.00020935
Glutathione metabolism	3.15 × 10^−5^	Sesquiterpenoid and triterpenoid biosynthesis	0.00306591
Drug metabolism–cytochrome P450	0.00019061	Butirosin and neomycin biosynthesis	0.00510528
Complement and coagulation cascades	0.00193363	Streptomycin biosynthesis	0.01120151
Adipocytokine signaling pathway	0.00221758	Pancreatic secretion	0.01418717

**Table 4:** Genes regulated by micromolar mercury and lead using RNA-seq and qRT-PCR analysis.

Gene Symbol	Fold Change (Mercury)		Fold Change (Lead)		Biological Process
	RNA-Seq	qRT-PCR	RNA-Seq	qRT-PCR	
Regulated by specific exposure to mercury					
*intl2*	16.43	12.22 ± 0.06			Signal transduction
*per2*	11.70	14.92 ± 0.21			Response to hydrogen peroxide
*cry5*	9.31	10.17 ± 0.15			DNA repair
*cybb*	3.72	3.10 ± 0.16			Oxidoreductase activity
*hamp1*	2.55	2.14 ± 0.30			Cellular iron ion homeostasis
*nyx*	−5.06	−5.56 ± 0.36			Neurological system process
*opn1sw1*	−3.74	−4.32 ± 0.32			Visual perception
Regulated by specific exposure to lead					
*sult1st5*			3.53	4.80 ± 0.12	Xenobiotic metabolic process
*rrad*			3.51	3.67 ± 0.03	Small GTPase-mediated signal transduction
*socs3b*			2.85	2.73 ± 0.21	Intracellular signal transduction
*hspb9*			2.78	2.49 ± 0.09	Response to stress
*gck*			−2.68	−3.92 ± 0.10	Glycolysis
Co-regulated by mercury and lead exposure					
*mt2*	28.01	24.04 ± 0.12	8.18	9.51 ± 0.13	Metal ion binding
*ctssb.1*	15.00	12.36 ± 0.18	5.56	2.88 ± 0.02	Proteolysis
*prdx1*	6.29	5.82 ± 0.24	3.97	4.19 ± 0.11	Peroxisome, antioxidant activity
*txn*	4.60	4.33 ± 0.06	4.08	4.52 ± 0.30	Antioxidant activity
*sqrdl*	4.53	5.21 ± 0.02	2.96	3.62 ± 0.08	Oxidoreductase activity
*tmprss13a*	4.05	3.61 ± 0.13	2.38	2.49 ± 0.06	Proteolysis
*socs3a*	4.03	6.57 ± 0.07	2.57	3.45 ± 0.19	Protein ubiquitination, intracellular signal transduction
*trpv6*	3.74	2.64 ± 0.07	2.82	2.65 ± 0.00	Calcium ion transmembrane transport
*abcb6a*	3.21	2.54 ± 0.46	2.58	3.66 ± 0.38	Transmembrane transport, ATP catabolic process
*gsr*	3.19	3.38 ± 0.08	2.58	2.23 ± 0.22	Glutathione metabolism, oxidoreductase activity
*hbz*	2.55	2.14 ± 0.01	3.06	4.03 ± 0.04	Oxygen transporter activity
*fads2*	−2.70	−2.85 ± 0.12	−2.75	−3.34 ± 0.08	Fatty acid biosynthetic process
*zgc:92590*	−9.13	−22.55 ± 0.16	−7.19	−9.24 ± 0.09	Protein digestion and absorption

## Data Availability

All data are available in the article and Supplementary Materials. In order to analyze the gene expression regulated by either mercury treatment or lead treatment or co-treatment, we relisted the expressions of twelve mercury-induced genes (including *intl2*, *per2*, *cry5*, *cybb*, *hamp1*, *nyx*, *opn1sw1*, *mt2*, *ctssb.1*, *prdx1*, *hbz*, and *fads2*) in Table 4 which are shown in our previous study [20]. Sequencing data for WT and mercury-treated embryos were deposited in the NCBI Sequence Read Archive under accession number SRP089827; data for lead-treated embryos were uploaded in our lab’s database at Institute of Microbiology, Guangdong Academy of Sciences (http://www.gdim.cn, accessed on 4 September 2024).

## Supplementary Materials

The following supporting information can be downloaded at https://www.mdpi.com/article/10.3390/ani14192877/s1. Figure S1: Standard curves for qRT-PCR primer pair; Figure S2: Boxplot displaying the distribution of RPKM between untreated control and lead-treated groups. A horizontal line in the box shows the median RPKM value; Figure S3: GO enrichment analysis of lead-regulated DEGs. DEGs are grouped into ‘Cellular Component’, ‘Molecular Function’, and ‘Biological Process’. Red and green rectangles indicate the percent of up- and down-regulated genes, respectively. The blue rectangle refers to the total percent of DEGs regulated by lead. Table S1: Information on the primers used for qRT-PCR; Table S2: Number of genes expressed with different abundance; Table S3: Genes regulated by lead treatment; Table S4: Enriched GO terms for lead-regulated genes; Table S5: Enriched pathways for lead-regulated genes; Table S6: Genes co-regulated by mercury and lead treatment.
