# Supplementary material for: Analysis of Differential Gene Expression under Acute Lead or Mercury Exposure in Larval Zebrafish Using RNA-Seq
Source: Animals (Basel). 2024 Oct 6;14(19):2877. doi: 10.3390/ani14192877 (PMC11475140; doi:10.3390/ani14192877)
Supplement: Supplementary file 1 [file animals-14-02877-s001.zip › Supplementary Figures.pdf]

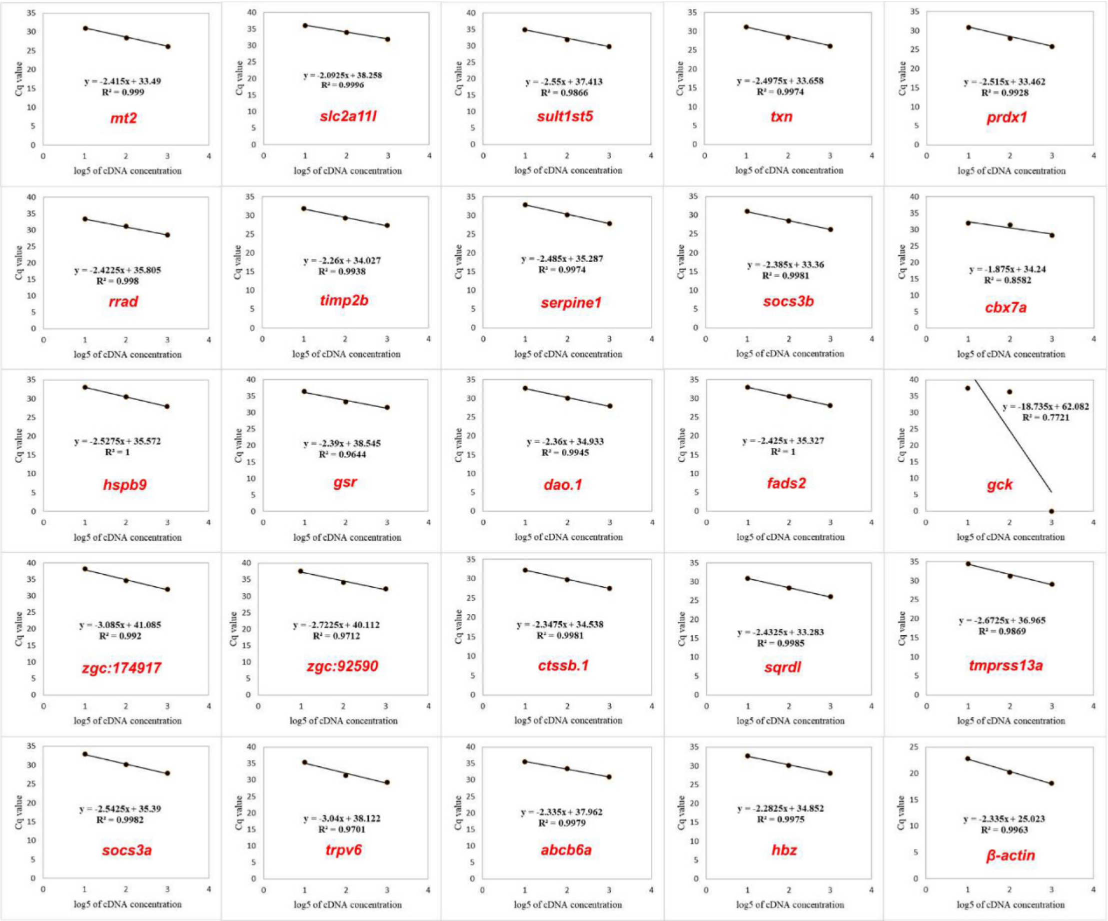

Figure S1. Standard curves for qPCR primer pair.

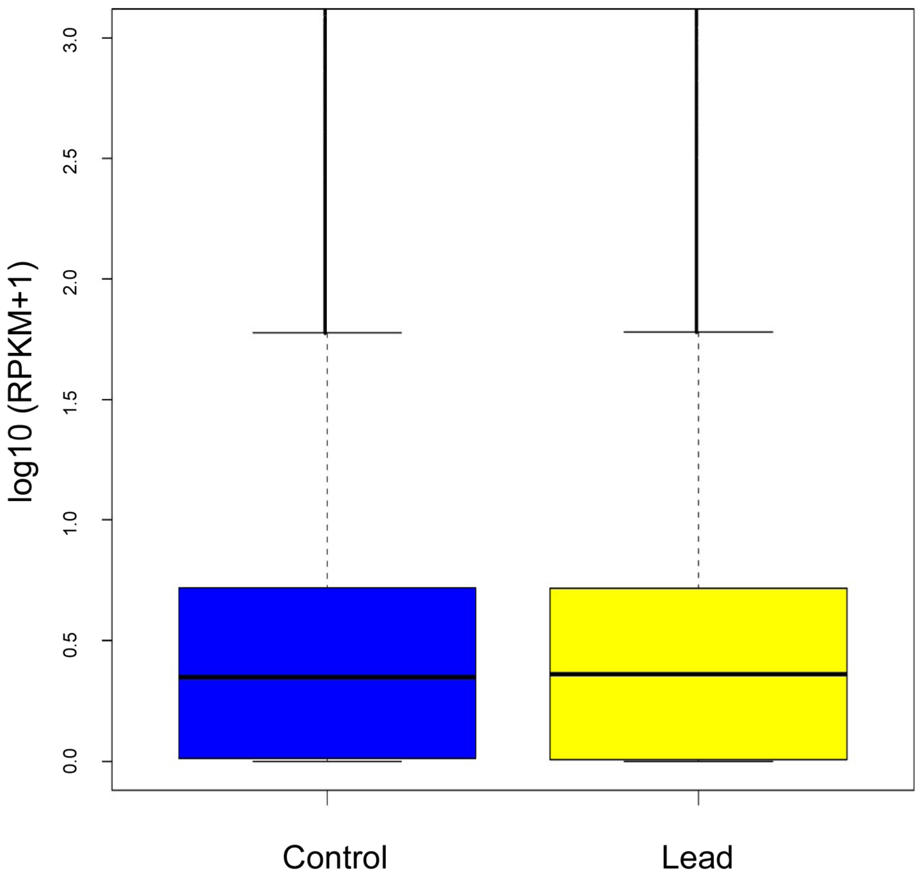

Figure S2. Boxplot displayed the distribution of RPKM between untreated control and lead-treated groups. A horizontal line in box shows the median RPKM value.

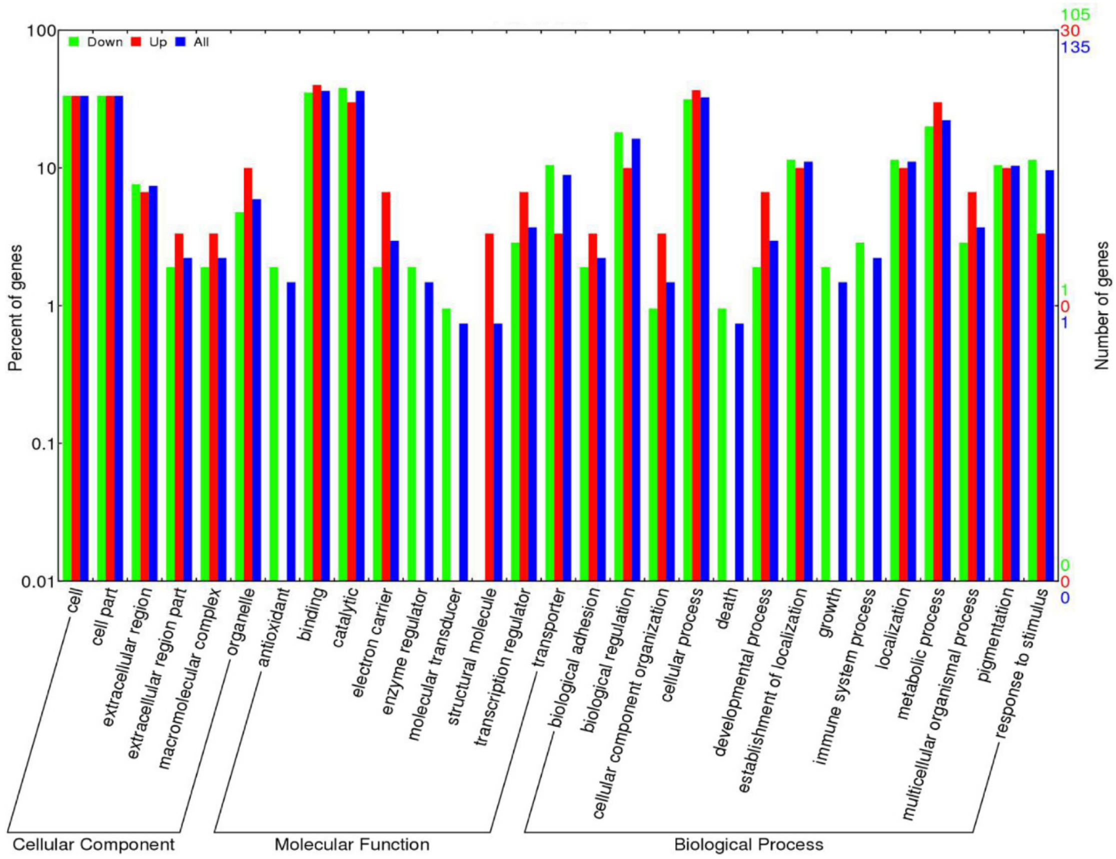

Figure S3. GO enrichment analysis of lead-regulated DEGs. DEGs were grouped into 'Cellular Component', 'Molecular Function', and 'Biological Process'. Red and green rectangles indicted the percent of up- and down-regulated genes, respectively. Blue rectangle refers to the total percent of DEGs regulated by lead.
